# Health economic evaluations comparing insulin glargine with NPH insulin in patients with type 1 diabetes: a systematic review

**DOI:** 10.1186/1478-7547-9-15

**Published:** 2011-10-06

**Authors:** Ernst-Günther Hagenmeyer, Katharina C Koltermann, Franz-Werner Dippel, Peter K Schädlich

**Affiliations:** 1 Fischzug 19H, 10245 Berlin, Germany; 2 Lychener Str. 45, 10437 Berlin, Germany; 3 Sanofi-Aventis Deutschland GmbH, Potsdamer Straße 8, 10785 Berlin, Germany; 4 IGES Institut GmbH, Friedrichstraße 180, 10117 Berlin, Germany

**Keywords:** Systematic review, health economics, type 1 diabetes, basal-bolus therapy, insulin glargine, NPH

## Abstract

**Background:**

Compared to conventional human basal insulin (neutral protamine Hagedorn; NPH) the long-acting analogue insulin glargine (GLA) is associated with a number of advantages regarding metabolic control, hypoglycaemic events and convenience. However, the unit costs of GLA exceed those of NPH. This study aims to systematically review the economic evidence comparing GLA with NPH in basal-bolus treatment (intensified conventional therapy; ICT) of type 1 diabetes in order to facilitate informed decision making in clinical practice and health policy.

**Methods:**

A systematic literature search was performed for the period of January 1st 2000 to December 1st 2009 via Embase, Medline, the Cochrane Library, the databases GMS (German Medical Science) and DAHTA (Deutsche Agentur für Health Technology Assessment), and the abstract books of relevant international scientific congresses. Retrieved studies were reviewed based on predefined inclusion criteria, methodological and quality aspects. In order to allow comparison between studies, currencies were converted using purchasing power parities (PPP).

**Results:**

A total of 7 health economic evaluations from 4 different countries fulfilled the predefined criteria: 6 modelling studies, all of them cost-utility analyses, and one claims data analysis with a cost-minimisation design. One cost-utility analysis showed dominance of GLA over NPH. The other 5 cost-utility analyses resulted in additional costs per quality adjusted life year (QALY) gained for GLA, ranging from € 3,859 to € 57,002 (incremental cost effectiveness ratio; ICER). The cost-minimisation analysis revealed lower annual diabetes-specific costs in favour of NPH from the perspective of the German Statutory Health Insurance (SHI).

**Conclusions:**

The incremental cost-utility-ratios (ICER) show favourable values for GLA with considerable variation. If a willingness-to-pay threshold of £ 30,000 (National Institute of Clinical Excellence, UK) is adopted, GLA is cost-effective in 4 of 6 cost utility analyses (CUA) included. Thus insulin glargine (GLA) seems to offer good value for money. Comparability between studies is limited because of methodological and country specific aspects. The results of this review underline that evaluation of insulin therapy should use evidence on efficacy of therapy from information synthesis. The concept of relating utility decrements to fear of hypoglycaemia is a plausible approach but needs further investigation. Also future evaluations of basal-bolus insulin therapy should include costs of consumables such as needles for insulin injection as well as test strips and lancets for blood glucose self monitoring.

## Background

The aim of diabetes therapy has always been to mimic the basal and mealtime components of endogenous insulin secretion. Since intensive conventional treatment (ICT) was introduced in the 1960s this was achieved by applying short-acting and intermediate-acting human insulin [1]. Throughout the 1990s insulin pumps with a programmable insulin secretion profile became increasingly available. As a third option the first synthetic long-acting insulin analogue insulin glargine (GLA) was approved by the European Medicines Agency (EMA) and Food and Drug Administration (FDA) in 2000 [2].

GLA is produced using a recombinant DNA technology. After injection GLA precipitates in the subcutaneous tissue and the absorption into the bloodstream is delayed. Pharmacodynamic studies showed that GLA covers the basal demand over 24 hours. It is closer to the physiological insulin release than intermediate-acting NPH insulin [3].

The efficacy of GLA has been extensively studied in type 1 diabetes. Three systematic reviews [4-6] and one meta-regression [7] cover this topic.

As type 1 diabetes is a lifelong condition starting in childhood, optimal metabolic control is very important to prevent disease specific micro- and macrovascular complications. In addition, the incidence of type 1 diabetes in children younger than 15 years is increasing in Europe, and thus the future burden of this disease. For 2020 the number of new cases in Europe is predicted to be 24,400 per annum. The prevalence of type 1 diabetes in children under 15 is expected to rise by 70% [8].

The unit cost of GLA is higher than that of conventionally used intermediate-acting NPH insulin. As all health care systems have to make optimal use of scarce resources, economic evaluation of GLA is an important issue. Because conduct and interpretation of economic evaluation is an extensive and complex effort a systematic review of the existing health economic evidence might be useful for many third party payers and other decision makers in health care.

The aim of the present study was to systematically review the published health economic evaluations comparing GLA with NPH as the basal component of an ICT in patients with type 1 diabetes.

## Methods

The design of the systematic review was based on the recommendations of the PRISMA Statement [9]. The following predefined criteria were applied for the inclusion of studies:

• patients with type 1 diabetes only; studies, where type 1 diabetes was mixed with type 2 diabetes or undefined diabetes types were excluded

• intervention with GLA as the basal component of intensified conventional therapy (ICT)

• NPH as comparator

• comparative health economic design: cost-minimisation analysis (CMA), cost-effectiveness analysis (CEA) cost-utility analysis (CUA) or systematic reviews about studies of the corresponding type

• at least one of the following parameters as target parameters: difference of treatment costs, incremental direct costs, incremental indirect costs, incremental cost-effectiveness ratio (ICER)

• full publication in English or German language between January 1st 2000 and December 1st 2009. If a full publication does not exist either a detailed study report has to be available or a congress paper, which contains all the necessary information for the quality evaluation and standardised data extraction. If the information covered by the congress paper is not sufficient, personal correspondence with the author has to provide all necessary information for the standardised data abstraction form.

The following electronic data bases were searched: Medline, Embase, Cochrane Library, National Health Service's Database of Abstracts of Reviews of Effects (NHS-DARE), National Health Service Economic Evaluation Database (NHS-HTA), as well as the German Medical Science Database (GMS) of the German Institute of Medical Documentation and Information (DIMDI). The database of DIMDI also included the database of the German Agency of Health Technology Assessment (DAHTA). For the respective search strings see Additional file 1. Additionally, a hand search in the German Diabetology journals was conducted for the years 2007 to 2009 as well as in abstract books of relevant international scientific congresses: the abstract databases of the Annual European respectively the Annual International Congresses of the International Society for Pharmacoeconomics and Outcomes Research (ISPOR), of the Annual Scientific Sessions of the American Diabetes Association (ADA), of the Annual International Meetings and the of the Annual Meetings of the European Association for the Study of Diabetes (EASD), and of the Annual Meeting of the German Diabetes Society (DDG) were scanned for relevant studies during the period of 2007 to 2009. The manufacturer of insulin glargine was asked to provide all relevant studies.

Two reviewers (KCK and EGH) independently selected publications for inclusion. Differences in decisions between the reviewers were resolved by consensus. Identified records were assessed in a two-stage procedure. First, title and abstract were screened for compliance with the defined inclusion criteria. All double publications were excluded, and in the case of doubt full text publications were obtained. In the second step, full texts of the remaining studies were assessed for inclusion.

After inclusion, the quality of the remaining studies was evaluated [10]. To assess the quality, widespread tools are available such as the tools of Leidl et al. [11], of Aidelsburger et al. [12], of Drummond and Jefferson [13] or Drummond et al. [14]. As a limitation, these checklists do not cover more recent aspects of health economic evaluation such as complex modelling. Also the requirements for health economic evaluation in diabetology are not considered. Economic analyses of diabetes mellitus treatment should consider the chronicity of the disease by a time horizon long enough to cover the broad spectrum of long-term consequences and their impact on quality of life [15]. Therefore, based on the publications above an extended checklist was developed (see Additional file 2).

For the evaluation of claims data analyses the quality characteristics of observational studies were applied, as they can be deduced from the STROBE Initiative [16]: description of setting and inclusion criteria of the study, definition of exposition and target parameter, confounding control, appropriate statistical analysis techniques, as well as a consistent presentation of results.

Key elements of the studies were captured in standardised abstraction forms either for modelling studies or for observational studies.

The ICER of the included studies were transferred into Euro values via purchasing power parities (PPP) for easier comparison, as proposed by Welte et al. [17] and Drummond et al. [18]. PPP values were obtained from the German Federal Statistical Office [19]. Euro values were calculated for the year of costing as used in the corresponding study (see Additional file 3).

## Results

**Figure 1:**
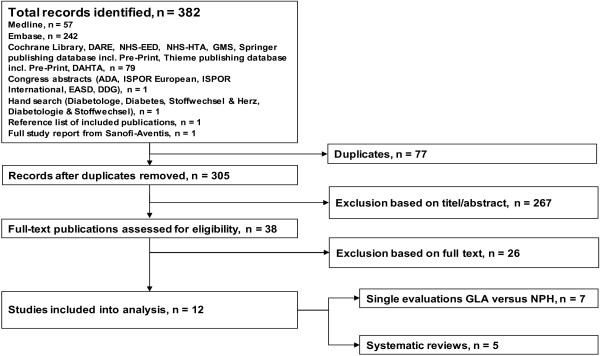
**Flow chart of study selection**. ADA = American Diabetes Association, DAHTA = Deutsche Agentur für Health Technology Assessment, DARE = National Health Service's Database of Abstracts of Reviews of Effects, DDG = Deutsche Diabetes Gesellschaft, EASD = European Association for the Study of Diabetes, GLA = Insulin glargine, GMS = German Medical Science, ISPOR = International Society for Pharmacoeconomics and Outcomes Research, NHS-EED = National Health Service Economic Evaluation Database, NHS-HTA = National Health Service Health Technology Assessment Database, NPH = Neutral Protamine Hagedorn insulin.

The search yielded 382 publications, 267 of which were excluded based on title and abstract screening. Following the full text review, a total of 12 published articles were selected for final inclusion (Figure 1): 6 modelling studies [20-25], 1 claims data analysis [26] and 5 systematic reviews [27-31].

Two of the identified studies were based on the evaluation of GLA by NICE [32] in the year 2002. One was the report of the assessment group on the primary model [20]. The other one was the detailed publication of the amended final model [24], which was the basis for the final appraisal in the Technical Appraisal Guidance by NICE [32].

The modelling studies were conducted in health care systems such as Canada [21,22], Great Britain [20,23,24] and Switzerland [25]. The claims data analysis [26] was conducted in the German setting.

### Modelling studies

Table 1 presents an overview of the 6 modelling studies [20-25] in detail. All of the studies were conducted as incremental cost-utility analyses.

**Table 1:** Main characteristics of modelling studies with GLA vs.NPH (listed in order of increasing ICER in €/QALY^a^)

Author/study (year) country/perspective/time horizon (discount rate) initiator	Type of economic evaluation/methodological approach	Effect of GLA on HbA1c compared to NPH	Effect of GLA on frequency of hypoglycaemia compared to NPH	Long-term complications of diabetes	Utilities	Results for GLA compared to NPH	ICERs in €/QALY^a^
**Brändle et al.**[25]							
Switzerland third party payer perspective 40 years (C 3.5%, E 3.5%) Sanofi-Aventis	CUA DES based on McEwan et al. [23] and DCCT	-0.19% points according to Mc Ewan [23]	Symptomatic: -23% Severe: -24% Nocturnal: -24% All reductions based on [7]	Reduction depending on HbA1c reduction	Reduction by: 1. hypoglycaemia 2. fear of hypoglycaemia 3. long-term consequences	**IU: **0.238 QALYs more **IC: **CHF 1,476 less **ICER: **GLA dominant	**dominant**
**McEwan et al. **[23] **Scenario 5**							
UK NHS 40 years (C 3.5%, E 3.5%) Sanofi-Aventis	CUA DES based on DCCT	-0.45% points^b^	-	Reduction depending on HbA1c reduction	Reduction by: 1. long-term consequences	**IU: **0.12 to 0.34 QALYs more **IC: **£ 1,043 to £ 1,371 more **ICER: **£ 1,096/QALY	€ 3,859
**Warren et al. **[24]							
UK NHS 9 years ( C 3.5%, E 3.5%) NICE	CUA ScHARR Model	Only in sensitivity analysis: -0.14% points [33]	Symptomatic: -42% [35] Severe: -52% [35]	In sensitivity analysis reduction depending on HbA1c reduction	Reduction by: 1. hypoglycaemia 2. fear of hypoglycaemia 3. long-term consequences only in sensitivity analysis	**IU: **n/a **IC: **£ 573 to £ 816 more **ICER: **£ 3,496 to £ 4,978 per QALY	€ 4,073 to € 5,800
**McEwan et al. **[23] **Scenario 1-3**							
UK NHS 40 years (C 3.5%, E 3.5%) Sanofi-Aventis	CUA DES based on DCCT	-	Severe: -25 to -28%^b^ Nocturnal: -17 to -22%^b^	-	Reduction by: 1. hypoglycaemia 2. fear of hypoglycaemia	**IU: **0.12 to 0.34 QALYs **IC: **£ 1,043 to £ 1,371 more **ICER: **£ 8,807 to £ 7,391 per QALY	€ 8,943 to € 10,656
**McEwan et al. **[23] **Scenario 4**							
UK NHS 40 years (C 3.5%, E 3.5%) Sanofi-Aventis	CUA DES based on DCCT	-0.19% points^b^	-	Reduction depending on HbA1c reduction	Reduction by: 1. long-term consequences	**IU: **0.12 to 0.34 QALYs more **IC: **about £ 1,043 to £ 1,371 more **ICER: **£ 1,096/QALY	€ 11,818
**Grima et al. **[22]							
Canada Canadian health ministry 36 years (C 5%, E 5%) Sanofi-Aventis	CUA State Transition Model based on UKPDS and DCCT	-0.4% points [34]	-	Reduction depending on HbA1c reduction	Reduction by: 1. long-term consequences	**IU: **0.08 QALYs more **IC: **CAN$ 1,398 more **ICER: **CAN$ 20,799/QALY	€ 13,364
**Warren et al. **[20]							
UK NHS 9 years (C 3.5%, E 3.5%) NICE	CUA ScHARR Model	Only in sensitivity analysis: -0.14% points [33]	Symptomatic: -19% [20] Severe: -52% [35]	Reduction depending on HbA1c reduction	Reduction by: 1. hypoglycaemia 2. fear of hypoglycaemia 3. long-term consequences only in sensitivity analysis	**IU: **n/a **IC: **£ 962 more **ICER: **£ 32,244/QALY	€ 37,567
**Cameron et al. **[21]							
Canada Canadian health ministry 60 years (C 5%, E 5%) CADTH	CUA based on CORE-Model	-0.11% points [5]	Moderate: -18% [5] Severe: -18% [5]	Reduction depending on HbA1c reduction	Reduction by: 1. hypoglycaemia 2. fear of hypoglycaemia only in sensitivity analysis 3. long-term consequences	**IU: **0.039 QALYs more **IC: **CAN$ 3,423 more **ICER: **CAN$ 87,932/QALY	€ 57,002

Legends: C = costs, E = effects, UK = United Kingdom, CADTH = Canadian Agency, CUA = Cost-Utility-Analysis, QALY = quality adjusted life-year, CORE = Centre for Outcomes Research, DES = discrete event simulation, NICE = National Institute for Health and Clinical Excellence, NHS = National Health Service, IU = incremental utilities, IC = incremental costs, ICER = incremental cost-effectiveness ratio, n/a = not applicable, ScHARR = School of Health and Related Research (University of Sheffield).

^a^Currencies transformed into Euro values via purchasing power parities (PPP), ^b^unpublished material

#### Quality assessment of modelling studies

The results of the quality assessment are given in Table 2.

**Table 2:** Quality characteristics of modelling studies

Author/Study (year of publication)	**Brändle et al. **[25]	**Cameron et al. **[21]	**McEwan et al. **[23]	**Grima et al. **[22]	**Warren et al. **[24]	**Warren et al. **[20]
Research question clearly defined	(✓)	(✓)	✓	✓	✓	✓
Perspective named	(✓)	(✓)	✓	✓	✓	✓
Considered health effects	HbA1c, hypoglycaemia, fear of hypogl.	HbA1c, hypoglycaemia, fear of hypogl.^1^	HbA1c, hypoglycaemia, fear of hypogl.	HbA1c	HbA1c^1^, hypoglycaemia, fear of hypogl.	HbA1c^1^, hypoglycaemia, fear of hypogl.
Source of efficacy data	Meta-analysis^2^, Meta-regression	Meta-analysis	Meta-analysis^2^	RCT	RCT	Meta-analysis, RCT
Determination of efficacy via information synthesis	-	✓	✓	-	-	-
Complications of diabetes considered	✓	✓	✓	✓	✓^1^	✓^1^
All relevant costs considered	✓	(✓)	(✓)	(✓)	(✓)	(✓)
Discounting (rate)	C (3,5%), E (3,5%)	C (5%), E (5%)	C (3,5%), E (3,5%)	C (5%), E (5%)	C (3,5%), E (3,5%)	C (3,5%), E (3,5%)
Incremental analysis	✓	✓	✓	✓	✓	✓
Sensitivity analysis	✓	✓	✓	✓	✓	✓
Results of sensitivity analysis described	✓	✓	✓	✓	✓	(✓)
Results presented per capita	✓	✓	✓	✓	✓	✓
Research question answered	✓	✓	✓	✓	✓	✓
Strength and weakness of studies discussed	-	✓	-	✓	(✓)	(✓)

Legends: C = cost, E = effectiveness, HbA1c = haemoglobin A1c, RCT = randomised controlled trial. ^1^Parameter included in sensitivity analysis only, ^2^unpublished material

All of the modelling studies considered long-term consequences of diabetes. The effectiveness data of 3 studies [20,22,24] were based on selected randomised controlled trials (RCTs [33-35]). The choice of the trial by Porcellati et al. was motivated by its comparatively large sample size and 12 months duration. The other RCTs are referred to as being representative. Warren et al. [20] used a meta-analysis done by themselves and one RCT [33]. One modelling study [21] used a recently published meta-analysis [5] which included 11 studies. Being most recent it should cover most of the available evidence. McEwan et al. [23] refer to an unpublished meta-analysis. They chose for their 5 scenarios different values from meta-analyses on 3 different subgroups of studies: all studies, studies of ≥ 3 months duration, pre-registration studies. Brändle et al. [25] used data from the above mentioned unpublished meta-analysis to determine the value of HbA1c reduction. They used data from meta-regression based on all available patient-level data from all randomized phase III and IV clinical trials sponsored by the manufacturer of GLA that compared GLA and NPH available in May 2004 [7] to determine the rates of hypoglycaemia reduction in relationship to glycaemic control. Individual patient data from other randomized phase III and IV clinical trials comparing GLA and NPH retrieved from MEDLINE, EMBASE, and BIOSIS were not available at that time [7]. This concept has been discussed and accepted by other authors [20,24].

Only the modelling study of Brändle et al. [25] considered needles, blood glucose test strips or lancets, which contribute significantly to insulin therapy costs. Two of the studies lack a complete description of therapy alternatives and of the perspective of the economic evaluation [21-25].

Most of the modelling studies were of good quality regarding incremental analysis, sensitivity analysis, description of general results, and presentation of results per capita as well as answering the research question. Despite the clear guidelines of NICE for economic analysis, the short descriptions of the models in the two studies linked to the NICE appraisal [20,24] made it difficult to understand the structure of the model, the input parameters, and especially the use of utility values. Furthermore, in the publication of Warren et al. [20] the description of the results of the sensitivity analysis was limited.

Overall the included modelling studies showed an acceptable or good quality. They had sufficient explanatory power assessing the cost-effectiveness of long-acting insulin analogue (GLA) versus the selected comparator (NPH).

#### Input parameters: clinical effects

Table 1 gives for every modelling study the sources for the parameters of clinical effectiveness. Most of the modelling studies assumed that under treatment with GLA compared to NPH either the metabolic adjustment improved under a comparable frequency of hypoglycaemic events, or the frequency of hypoglycaemia decreased under comparable metabolic control. Accordingly, both studies of Warren et al. [20,24] only considered a reduction of symptomatic hypoglycaemia (-19% and -42%, respectively) and severe hypoglycaemia (both -52%). In sensitivity analyses these models used an additional HbA1c reduction of 0.14%-points under GLA without reduction of hypoglycaemia. Grima et al. [22] only used an additional reduction of HbA1c of 0.4% points under GLA. In a more advanced approach, McEwan et al. [23] made use of unpublished meta-analyses with different scenarios. In scenario 1 to 3, the risk of severe hypoglycaemia was reduced between 25 and 28% and the risk of nocturnal hypoglycaemia between 17 to 22% under GLA. In scenario 4 and 5, HbA1c was reduced under GLA additionally by 0.19% points and 0.45% points, respectively, without changing the rate of hypoglycaemia.

Combined effects on hypoglycaemia and on metabolic control were implemented in two studies: Cameron et al. [21] used meta-analyses [5] for reduction of moderate and severe hypoglycaemia (both -18%) combined with an HbA1c reduction of 0.11% points. In the model of Brändle et al. [25] a reduction of severe (-24%), nocturnal (-24%) and symptomatic (-23%) hypoglycaemia was combined with a HbA1c reduction of 0.19%-points.

#### Input parameters: utilities

Quality of life is reduced by diabetes-related long-term macro- and microvascular complications such as coronary heart disease comprising angina pectoris, myocardial infarction, congestive heart failure, and nephropathy as well as retinopathy [36]. Different approaches were used in the included studies as shown in Table 3. Grima et al. [22] used utilities for the different long-term consequences of diabetes. In the case of several coexisting complications the lowest applicable utility was used. McEwan et al. [23], Brändle et al. [25] and Cameron and Bennett [21] used utility decrements, summing up different coexisting diabetic long-term consequences and hypoglycaemia events.

**Table 3:** Utilities and utility decrements used in modelling studies

Author/Study	**Cameron et al**. [21]	**Grima et al**. [22]	**McEwan et al**. [23]	**Brändle et al**. [25]	**Warren et al**. [24]	**Warren et al**. [20]
**Health state**	**Utility decrement^a^**	**Utility values^f^**	**Utility decrement^a^**	**Utility decrement^a^**	**Utility decrement^a^** **Data except for hypoglycaemia confidential to NICE**	

Ischaemic heart disease, first & subsequent years	-	-	-0.09	-0.09	n/a	n/a
Myocardial infarction, year of event	-0.0409222	0.760	-0.066	-0.055	n/a	n/a
Myocardial infarction, subsequent years	-0.012	0.800	-0.066	-0.055	n/a	n/a
Angina, year of event	-0.0411989	-	-	-	n/a	n/a
Angina, subsequent years	-0.024	-	-	-	n/a	n/a
Congestive heart failure, year of event	-0.0546	0.693	-0.108	-0.108	n/a	n/a
Congestive heart failure, subsequent years	-0.018	0.693	-0.108	-0.108	n/a	n/a
Stroke, year of event	-0.0523513	0.720	-0.114	-0.164	n/a	n/a
Stroke, subsequent years	-0.040	0.800	-0.114	-0.164	n/a	n/a
Peripheral vascular disease	-0.021	-	-	-	n/a	n/a
Microalbuminuria	-0.012	-	-	-	n/a	n/a
Proteinuria	-0.017	-	-	-	n/a	n/a
ESRD, first and subsequent years	-	0.644	-0.263	-0.263	n/a	n/a
Dialysis, first & subsequent years	-0.16	-	-0.305	-0.305	n/a	n/a
Funct. kidney transplant, first & subsequent years	-0.03	-	-0.075	-0.075	n/a	n/a
Diabetic retinopathy	-0.0155836	0.750	-	-	n/a	n/a
Blindness and low vision	-0.0497859	-	-	-	n/a	n/a
Pre-blind, first & subsequent years	-	-	-0.029	-0.029	n/a	n/a
Blind, first & subsequent years	-	-	-0.074	-0.074	n/a	n/a
Cataract	-0.0170832	-	-	-	n/a	n/a
Macular edema	-0.0170832	-	-	-	n/a	n/a
Neuropathy	-0.0243702	-	-	-	n/a	n/a
Active uninfected diabetic ulcer	-0.09	-	-	-	n/a	n/a
Active infected diabetic ulcer	-0.14	-	-	-	n/a	n/a
Amputation	-0.266	0.678	-0.28	-0.28	n/a	n/a
Amputation, subsequent years	-	0.800	-	-	n/a	n/a
Fear of any hypoglycaemic episode	-	-	-	-	-0.0019^d^	-0.0052^d^
Severe hypoglycaemic episode	-0.5485^b^	-	-0.047^d^	-0.047^d^	-0.15^e^	-0.15^e^
Mild/moderate hypoglycaemic episode	-0.167^c^	-	-	-	-	-
Symptomatic hypoglycaemic episode	-	-	-0.0142^d^	-0.0142^d^	-	-
Nocturnal hypoglycaemic episode	-	-	-0.0084^d^	-0.0084^d^	-	-

Legend: ESRD = endstage renal disease

^a^applied for 1-year (but not for subsequent years, hypoglycemic episodes and ulcers)

^b^decrement for 24 hours

^c^decrement for 15 minutes

^d^unclear duration

^e^decrement for 4 days

^f^not additive, lowest utility in respective year is used

In general the utility decrements for long-term complications used by McEwan et al. [23] and Brändle et al. [25] are higher than those used by Cameron and Bennett [21] (Table 3).

Significant methodological differences exist in dealing with the influence of hypoglycaemic events on quality of life. The following utility decrements were attributed by Cameron and Bennett [21] to hypoglycaemic events: 0.0015 per severe and 0.0000048 per mild/moderate hypoglycaemia and year.

Warren et al. [20,24], McEwan et al. [23] and Brändle et al. [25] went even further and assumed, that quality of life is not only affected by hypoglycaemia itself, but also by a longer-lasting fear of this event. The derivation of utility reduction was described by Currie et al. [37]. Patients with type 1 diabetes were asked about frequency and severity of hypoglycemic events during the last 3 months as well as about their quality of life (via EQ-5D) and their fear of hypoglycaemia (via Hypoglycaemia Fear Score, HFS). Frequency and severity of hypoglycaemia were connected to the HFS by means of regression models and afterwards the HFS was linked to the EQ-5D values. For severe hypoglycaemia the utility decrement was 0.047 per event, for symptomatic 0.0142 and nocturnal 0.0084, respectively. The duration of the decrement remained unclear [37].

#### Input parameters: cost per unit consumed

It can be assumed that health care costs of diabetic long-term consequences and hypoglycemic events were considered properly in the included modelling studies. Except Warren et al. [20,24] all studies report unit costs and their sources for diabetic long-term complications.

The mean daily cost for GLA and NPH were not reported in the studies of Warren et al. [20,24] and Brändle et al. [25]. In the other studies the ratio of daily insulin cost between GLA and NPH varies between 1.81 [23], 2.24 [22] and 2.53 [21]. As different insulin costs are likely to have an impact on the results they should have been made transparent.

As shown in a claims data analysis [26], the costs of needles, test strips or lancets significantly influence the cost of diabetes care, it is important to mention, that only the study of Brändle et al. [25] accounted for these costs.

#### Structure parameters of the models

All models discounted not only the costs arising in the future but also the effects. Grima et al. [22] and Cameron and Bennett [21] used a discount rate of 5%, the remaining studies 3.5%. In the two studies of Warren et al. [20,24] a time horizon of 9 years was chosen for modelling. In the other studies the horizon was 36 [22], 40 [23,25][ and 60 years [21].

#### Outcome parameter incremental cost-effectiveness ratio

In Table 1 the identified modelling studies are presented in ascending order of their ICER value in purchasing power parities (PPP).

A strict coherence between the characteristics of the models and the ICER value cannot be deduced from this evaluation. But there are plausible explanations for the position of the respective study in the ranking order of the table.

GLA was dominant compared to NPH in the study from Brändle et al. [25]. Four aspects may have contributed to this favourable result: (i) the authors utilised both the positive impact of the GLA therapy on the metabolic control as well as on the frequency of hypoglycaemia; (ii) it is the only modelling study in this review that accounted for the costs of needles for insulin injection and disposables for blood glucose self-monitoring; (iii) utility decrements following the concept of fear of hypoglycaemia were applied; (iv) furthermore Brändle et al. [25] as well as McEwan et al. [23] used relatively high utility decrements compared to Cameron und Bennett [21]. The smallest ICER of € 3,859 per QALY gained is the result of scenario 5 from McEwan et al. [23]. In this model, which is a predecessor of the one Brändle et al. [25] used, a comparably high value for the HbA1c reduction of 0.45% points was applied; the frequency of hypoglycaemia was assumed to be the same for GLA and NPH.

In the studies on position 3 and 4, only a reduced frequency of hypoglycaemia under GLA was considered. Warren et al. [24] on position 3 used a reduced frequency of symptomatic hypoglycaemia by 42% and of severe by 52%, which are the highest reductions identified in this review. Compared to this McEwan et al. [23] with scenario 1-3 used lower values: frequency of symptomatic hypoglycaemia was reduced by 25-28% and of nocturnal by 17-22%. Both studies apply the concept of utility decrements related to the fear of hypoglycaemia. The ICERs range between € 4,073 per QALY gained [24] and € 10,565 in scenario 1-3 [23].

An ICER of € 11,818 per QALY gained was the result of the calculations in scenario 4 of McEwan et al. [23]. In contrast to scenario 5, the authors adopted a conservative value of the additional HbA1c reduction under GLA by 0.19% points. The frequency of hypoglycaemia is maintained equal for GLA and NPH.

Grima et al. [22] only used an additional HbA1c reduction of 0.40% points under GLA in their model, resulting in an ICER of € 13,364 per QALY gained.

The next higher ICER of € 37,567 per QALY gained was calculated with the earlier version of the ScHARR model [20]. Compared to [24] it used more conservative values for the reduction of hypoglycaemic events (symptomatic -20%/severe -52%) and also for the utility decrement related to fear of hypoglycaemia (-0.0019 versus -0.0052 per event).

The highest ICER value of € 57,003 per QALY gained resulted from the study of Cameron und Bennett [21]. GLA showed an advantage in the HbA1c reduction (-0.11% points) as well as in the lower rate of hypoglycaemia (moderate -18%/severe -18%) over NPH. The discount rate used was 5%. Utilities were not decreased by the fear of hypoglycaemia. Overall the utility decrements were lower than those used by McEwan et al. [23] and Brändle et al. [25] (see table 3).

### Claims data analysis

One claims data analysis [26] comparing GLA to NPH was included in the evaluation. It has not yet been published but was made accessible by the sponsor as full study report. This retrospective cohort study was done using German Statutory Health Insurance (SHI) claims data of type 1 diabetes patients treated with GLA (n = 656) or NPH (n = 638) in a cost-minimisation study.

#### Quality assessment of claims data analysis

Analyses of claims data are retrospective observational evaluations. Therefore, we applied different quality criteria compared to the modelling studies [16].

The included claims data analysis showed good quality concerning the description of main characteristics of the study design and the observed population. Also, confounder control via propensity score matching, the use of non-parametric tests for statistical analyses, and the description of the results were classified as adequate. Due to incompleteness of data, no costs of needles and lancets were calculated. The documentation of unit costs of insulin and of test strips used is missing in the report.

#### Outcome parameters

The average costs of all diabetes-specific outpatient prescriptions (long- and short-acting insulins, test strips) in the 15 months period were € 200 higher in patients with GLA than in NPH patients (p < 0.001). Patients treated with GLA consumed less but more expensive long-acting insulin (Δ € 124; p < 0.001) as well as more and costlier short-acting insulin (Δ € 63; p < 0.001). No difference was found in the consumption and costs of test strips.

No difference could be identified in utilization of acute hospital and emergency services, which was interpreted as evidence that there was no difference in effectiveness between both treatment strategies. Unfortunately, the evaluation did not account for the utilisation of insulin needles and lancets due to lack of data.

## Discussion

We conducted a systematic review of health economic evaluations comparing GLA versus NPH as the basal component of an ICT in type 1 diabetes. 7 economic evaluations from 4 different countries (Germany, Canada, England, Switzerland) were included: 6 cost-utility analyses based on complex modelling and 1 cost-comparison analysis based on claims data. In 1 cost-utility analysis GLA was dominant over NPH due to 0.238 additional QALYs gained together with cost savings of € 796 (time horizon 40 years). In the other 5 studies of this type additional costs per QALY gained for treatment with GLA ranged between € 3,859 and € 57,002.

There is no unique willingness-to-pay threshold for a QALY across different countries. However NICE judges a technology acceptable if the ICER is below £ 20,000 to £ 30,000 (€ 23,577 to € 35,365, based on 2009 PPP values) [38] and there are other statements that imply comparable threshold values for other countries [39]. Taking the upper threshold value into account, GLA would be judged cost-effective in 4 of the 6 of CUAs identified.

The cost-comparison analysis in the German SHI setting showed € 160 higher diabetes-specific costs per patient per 12 months for therapy with GLA compared to NPH.

The identified systematic reviews [27-31] only gave little detail on health economic evaluations comparing GLA versus NPH, all of them dealing with the GLA-NPH comparison among several other interventions related to type 1 diabetes. These reviews identified no additional studies compared to our search and reported no additional aspects.

Keeping in mind the challenges associated with modelling a chronic disease such as type 1 diabetes the methods of health economic evaluation are highly developed in this field of comparing different strategies of insulin therapy.

Overall the assessment of the quality of the studies using standardised check lists revealed acceptable to good quality of the included studies. General guidelines and recommendations on health economic evaluations [13,14,40] emphasise, that publications must be optimally transparent about the model's structure, the input data, the algorithms used and the assumptions made in the study. In a minority of publications the structure of the model used could only be assumed. More transparency is necessary in the presentation of unit costs. Especially precise information on unit prices of the compared insulins was often missing.

More diligence should be spent on the presentation of the utilities used. This is of paramount importance, because these factors have a strong impact on the total results of a cost-utility analysis. In some studies the period corresponding to utility decrements incurred by hypoglycaemia remained unclear or could only be determined from other referenced articles. Furthermore, when comparing utility values for the same type of event between different studies (Table 3), we found considerable differences. These differences pose a challenge to the comparison of economic evaluations. Our approach to coping with this issue was to make the differences transparent as shown in Table 3.

In some publications a clear research question and the perspective of the health economic evaluation was missing. Also the discussion of strengths and weaknesses was not always satisfying.

The parameters of clinical effectiveness should be obtained from meta-analyses that include all existing clinical evidence [10,41].This has only been realised by Brändle et al. [25], McEwan et al. [23] Cameron et al. [21] and partly by Warren et al. [20]. Choice of a single RCT from the pool of existing studies by the other evaluations was weakly motivated. Information synthesis would have been possible, because already in 2002, when the first evaluations were done, several studies comparing GLA with NPH in type 1 diabetes did exist.

Only 1-Brändle et al. [25]-out of 6 modelling studies included cost of self-monitoring of blood glucose, which is a substantial cost in insulin treatment (about 30% of all prescription costs [26]).

Specifically for modelling of diabetes a consensus panel of the American Diabetes Association (ADA) [15] has developed recommendations, among which the most important are:

- As diabetes affects multiple organ systems, the models must include a wide range of complications.

- These complications of diabetes may take years or decades to occur, therefore the time horizon of the models must be sufficiently long.

- Because some of the diabetes complications greatly reduce a person's quality of life, this type of outcome should be considered in any analysis. Cost-utility analysis is the appropriate evaluation type for this.

These commonly accepted requirements for diabetes models were fulfilled by all of the included modelling studies.

However, the following issues are still under discussion regarding diabetes models and long-acting insulin analogues:

- The question has not been finally answered whether the therapy with long-acting insulin analogues predominantly affects frequency of hypoglycaemia or predominantly affects metabolic control. Also a combination of both effects seems possible. However all three possibilities should be considered in a modelling study by different scenarios based on adequate meta-analyses. Another option is the use of results from an individual patient data (IPD) meta-regression [7] as Brändle et al. [25] did. Still the relationship between the effect of GLA on HbA1c and on the frequency of hypoglycaemia as well as the use of this relationship in the economic model are not sufficiently transparent.

- The concept of fear of hypoglycaemia, which influences quality of life beyond the event of hypoglycaemia itself seems plausible. Though, there are only few data available [37] and research on this should be improved. In economic evaluation, results of alternatives with and without utility reduction because of fear of hypoglycaemia should be clearly distinguishable.

- Until now, only few modelling studies consider the differences in the consumption of needles, test strips for blood glucose self-monitoring and lancets between the different basal insulins in type 1 diabetes.

This last point clearly shows, that claims data analyses and primary data collection of insulin consumption, test strips, needles, and lancets are a useful and necessary supplement to modelling studies. They provide the data of real life resource consumption, which in the models may be linked to clinical effectiveness and patient reported outcomes (PRO) data.

The aim of this systematic review was to make the results of the included evaluations comparable via different methodical steps. First, all relevant information about design, analysis and modelling techniques, input and output parameters were extracted by standardised checklists. Second, study quality was consistently evaluated by an internationally standardised tool. Finally, costs per QALY gained were converted into Euro using the purchasing power parities. This was necessary in order to express different values of different studies in different countries in comparable Euro values. The reference year of the original analysis was maintained.

The study used published PPPs that were derived from a general basket of goods and services. For the use in health economic evaluations a basket specifically of health care goods, e.g. drugs and supplies, and services, e.g. ambulatory and inpatient care services, may be more appropriate. However, such a health care related basket of goods and services does not yet exist [17].

Other reasons may as well restrict the comparability of the included studies:

- The different economic evaluations are based on different health care settings and legislations, e.g. the Canadian Medicare system, the National Health Service (NHS) in the United Kingdom or the German Statutory Health Insurance setting.

- The information about the economic evaluations was not presented comparably transparent in all publications.

- One publication was identified in compliance with the pre-defined inclusion criteria from a congress abstract database [25]. Data for the review was obtained form the congress poster and by extensive personal correspondence with the author. The inclusion of work published in an international scientific congress seemed justifiable in the rapidly evolving research area of health economic evaluation.

## Conclusions

Six health economic modelling studies and 1 claims data analysis comparing insulin glargine to NPH in type 1 diabetes has been analysed.

The incremental cost utility ratios (ICER) vary from dominance to € 57,002, which are less acceptable to a health care decision maker. Despite some limitations concerning comparability mainly resulting from methodological and country specific aspects, insulin glargine (GLA) seems to offer good value for money compared to conventional human insulin (NPH) in patients with type 1 diabetes treated with a basal bolus regimen.

Comparability between studies is limited. Still the results of this review underline the importance of the following issues in economic evaluation of insulin therapy: evidence on efficacy of therapy from information synthesis, i.e. meta-analysis or meta-regression, should be used. Quality of life plays an important role in the evaluations and maximum transparency on the utilities applied is necessary. The concept of utility decrements from fear of hypoglycaemia is plausible and should be investigated further. Future evaluations of insulin therapy should include consumption of consumables for insulin injection and blood glucose self monitoring.

## Competing interests

EGH, KCK and PKS have worked on several research projects of IGES Institut GmbH that have been funded by Sanofi-Aventis Deutschland GmbH. FWD is an employee of Sanofi-Aventis Deutschland GmbH. EGH and KCK were employees of IGES Institut GmbH at the time of the conduct of the review and writing of the manuscript. PKS is an employee of IGES Institut GmbH.

## Authors' contributions

EGH conceived the review, composed the design and was in charge of the coordination of the study. EGH and KCK performed the filter screening of the identified publications and drafted the manuscript. KCK carried out the literature search and was responsible for the personal communication with authors of relevant studies if necessary. PKS and FWD contributed to the study design and to drafting the manuscript. All authors read and approved the final manuscript.

## Supplementary Material

Additional file 1 **Search strategy used in the systematic review**. Click here for file

Additional file 2 **Conversion of different currencies via PPPs into Euro values**. Click here for file

Additional file 3 **Checklist quality of health economic modelling studies**. Click here for file
