# Supplementary material for: Health economic evaluations comparing insulin glargine with NPH insulin in patients with type 1 diabetes: a systematic review
Source: Cost Eff Resour Alloc. 2011 Oct 6;9:15. doi: 10.1186/1478-7547-9-15 (PMC3200149; doi:10.1186/1478-7547-9-15)
Supplement: Additional file 1 — Search strategy used in the systematic review. [file 1478-7547-9-15-S1.DOCX]

APPENDIX 1

Search Strategy Medline

OVID Medline January 1st 2000 to present; searched December 1st 2009

Table 1: Search string for Medline

| Search number | Search string |
| --- | --- |
| 1 | Search "glargine "[Substance Name] |
| 2 | Search ("Economics"[Mesh] OR "Economics, Pharmaceutical"[Mesh] OR "Health Care Economics and Organizations"[Mesh] OR "Economics, Nursing"[Mesh] OR "Economics, Medical"[Mesh] OR "Economics, Hospital"[Mesh]) |
| 3 | Search "Costs and Cost Analysis"[Mesh] |
| 4 | Search "Cost-Benefit Analysis"[Mesh] |
| 5 | Search "Cost of Illness"[Mesh] |
| 6 | Search "Health Care Costs"[Mesh] OR "Health Expenditures"[Mesh] |
| 7 | Search 6 or 5 or 4 or 3 or 2 |
| 8 | Search 1 AND 7 |
| 9 | Search "Cost Savings"[Mesh] |
| 10 | Search 6 or 5 or 4 or 3 or 2 or 9 |
| 11 | Search 1 AND 10 |
| 12 | Search 8 AND (9 or 6 or 5 or 4 or 3 or 2) |

Search Strategy Embase

Embase January 1st 2000 to present; searched December 1st 2009

Table 2: Search strings for Embase

| Search number | Search string |
| --- | --- |
| [1](http://www.ncbi.nlm.nih.gov/sites/?querykey=14&dbase=pubmed&querytype=eSearch&) | Lantus.mp. |
| [2](http://www.ncbi.nlm.nih.gov/sites/?querykey=13&dbase=pubmed&querytype=eSearch&) | HOE901.mp. |
| [3](http://www.ncbi.nlm.nih.gov/sites/?querykey=12&dbase=pubmed&querytype=eSearch&) | insulin glargine/ |
| [4](http://www.ncbi.nlm.nih.gov/sites/?querykey=8&dbase=pubmed&querytype=eSearch&) | 1 or 2 or 3 |
| [5](http://www.ncbi.nlm.nih.gov/sites/?querykey=7&dbase=pubmed&querytype=eSearch&) | "cost"/ |
| [6](http://www.ncbi.nlm.nih.gov/sites/?querykey=6&dbase=pubmed&querytype=eSearch&) | "cost benefit analysis"/ |
| 7 | "cost of illness"/ |
| [8](http://www.ncbi.nlm.nih.gov/sites/?querykey=4&dbase=pubmed&querytype=eSearch&) | "cost effectiveness analysis"/ |
| [9](http://www.ncbi.nlm.nih.gov/sites/?querykey=3&dbase=pubmed&querytype=eSearch&) | "cost minimization analysis"/ |
| [10](http://www.ncbi.nlm.nih.gov/sites/?querykey=2&dbase=pubmed&querytype=eSearch&) | "cost utility analysis"/ |
| [11](http://www.ncbi.nlm.nih.gov/sites/?querykey=1&dbase=pubmed&querytype=eSearch&) | "health care cost"/ |
| 12 | pharmacoeconomics/ |
| 13 | health economics/ |
| 14 | "cost control"/ |
| 15 | 5 or 6 or 7 or 8 or 9 or 10 or 11 or 12 or 13 or 14 |
| 16 | 4 and 15 |
| 17 | limit 16 to yr="2000 -Current" |

Search Strategy DIMDI

DIMDI January 1st 2000 to present; searched December 1st 2009

Table 3: Search string for DIMDI

| Search number | Search string |
| --- | --- |
| 1 | CDAR94 CDSR93 DAHTA GA03 GM03 INAHTA SP97 SPPP TV01 TVPP |
| 2 | glargine* |
| 3 | cost* or econom* |
| 4 | 2 AND 3 |
