# Supplementary material for: Health economic evaluations comparing insulin glargine with NPH insulin in patients with type 1 diabetes: a systematic review
Source: Cost Eff Resour Alloc. 2011 Oct 6;9:15. doi: 10.1186/1478-7547-9-15 (PMC3200149; doi:10.1186/1478-7547-9-15)

## Appendix 2: Conversion of different currencies via PPPs (Purchasing Power Parities) into Euro values

Table 1: PPP values used

| Country | year | PPP |
| --- | --- | --- |
| Canada | 2005 | 1,5564 CAD/€ |
|  | 2007 | 1,5426 CAD/€ |
| United Kingdom | 2002 | 0,8583 £/€ |
|  | 2005 | 0,8265 £/€ |
|  | 2009 | 0,8483 £/€ |
| USA | 2009 | 1,3693 $/€ |

CAD = Canadian dollars, £ = Great Britain pounds, $ = US dollars, PPP = Purchasing Power Parities

Different currencies were converted to Euros using the following formula:


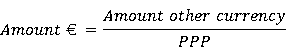

Supplement: Additional file 2 — Conversion of different currencies via PPPs into Euro values. [file 1478-7547-9-15-S2.DOC]
