# Supplementary material for: Health economic evaluations comparing insulin glargine with NPH insulin in patients with type 1 diabetes: a systematic review
Source: Cost Eff Resour Alloc. 2011 Oct 6;9:15. doi: 10.1186/1478-7547-9-15 (PMC3200149; doi:10.1186/1478-7547-9-15)
Supplement: Additional file 3 — Checklist quality of health economic modelling studies. [file 1478-7547-9-15-S3.DOC]

**Appendix 3: Checklist Quality of Health Economic Modelling Studies**

|  | | yes | partly | no | n/a | insufficient information given |
| --- | --- | --- | --- | --- | --- | --- |
| **Study question** | | | | | | |
| 1 | Was a clear study question posed (patient population, intervention, setting of care)? |  |  |  |  |  |
| 2 | Did the study involve a comparison of alternatives? |  |  |  |  |  |
| 3 | Was a comprehensive description of the competing alternatives given? |  |  |  |  |  |
| 4 | Was the chosen perspective for the analysis clearly defined? |  |  |  |  |  |
| **Study design** | | | | | | |
| 5 | Did the study examine both costs and effects of the intervention? |  |  |  |  |  |
| 6 | Is the type of economic evaluation stated? |  |  |  |  |  |
| 7 | Does the type of economic evaluation fit with the study question? |  |  |  |  |  |
| 8 | If the design of cost-minimisation anaysis was chosen: was the reason given for omitting health effects? |  |  |  |  |  |
| 9 | Was an appropriate time frame chosen for the study? |  |  |  |  |  |
| **Health effects** | | | | | | |
| 10 | Is the source of the data on effectiveness of the intervention given? |  |  |  |  |  |
| 11 | Are sufficient details reported on design and results of the studies on effectiveness? |  |  |  |  |  |
| 12 | Were randomised, controlled trials the source of the effectiveness data? |  |  |  |  |  |
| 13 | Was data on the effectiveness of the interventions based on observational studies or on assumptions? |  |  |  |  |  |
| 14 | Was effectiveness established through an information synthesis (systematic review, meta-analysis, meta-regression)? |  |  |  |  |  |
| 15 | Was the origin of data on health related quality of life reported in a transparent way? |  |  |  |  |  |
| 16 | Is the data on effectiveness transferable to the decision-making context? |  |  |  |  |  |
| 17 | Were all relevant health effects considered in the study? |  |  |  |  |  |
| **Costs** | | | | | | |
| 18 | Were all relevant costs considered in the study? |  |  |  |  |  |
| 19 | Was determination of utilisation of resources done in a valid way? |  |  |  |  |  |
| 20 | Were unit costs determined in a valid way? |  |  |  |  |  |
| 21 | Were costs and resource utilisation reported in a transparent way? |  |  |  |  |  |
| **Modelling** | | | | | | |
| 22 | Were the structure of the model and all used parameters made transparent to the reader? |  |  |  |  |  |
| 23 | Was the structure of the model adequate for answering the study question? |  |  |  |  |  |
| 24 | Were assumptions about model parameters adequate for the study context? |  |  |  |  |  |
| **Differential timing** | | | | | | |
| 25 | Were health effects as well as costs discounted? |  |  |  |  |  |
| 26 | Are the discount rates reported? |  |  |  |  |  |
| 27 | Are reasons given for the choice of the values of the discount rates? |  |  |  |  |  |
| **Incremental analysis** | | | | | | |
| 28 | Was an incremental analysis of costs and consequences of alternatives performed? |  |  |  |  |  |
| **Sensitivity analysis** | | | | | | |
| 29 | Was a sensitivity analysis performed? |  |  |  |  |  |
| 30 | Are the methods of the sensitivity analysis, varied parameters and ranges for variation of parameters reported? |  |  |  |  |  |
| 31 | Are the methods of the sensitivity analysis, choice of parameters and ranges for variation appropriate for answering the study question? |  |  |  |  |  |
| 32 | Are the results of sensitivity analyses reported comprehensively? |  |  |  |  |  |

|  | | yes | partly | no | n/a | insufficient information given |
| --- | --- | --- | --- | --- | --- | --- |
| **Presentation of results** | | | | | | |
| 33 | Are total life-time costs, total life expectancy and total quality adjusted life expectancy reported? |  |  |  |  |  |
| 34 | Are incremental health effects and incremental costs reported? |  |  |  |  |  |
| 35 | Is an answer to the study question given? |  |  |  |  |  |
| **Discussion** | | | | | | |
| 36 | Are the results compared to those of other studies with comparable study questions? |  |  |  |  |  |
| 37 | Are limitations of the study being discussed? |  |  |  |  |  |
